# The Bedding and Linen Department—V

**Published:** 1908-05-30

**Authors:** 


### Nursing Administration.

### THE BEDDING AND LINEN DEPARTMENT.

### V.—THE COST OF WASHING.

It is exceedingly difficult to ascertain what is spent on the laundry in one hospital as compared with another. Very few hospitals, for instance, deduct the cost of laundry for the nurses from the main account, though it would seem that this would not be difficult to do. For the most part, too, when there is a private laundry, as is now very usual, no attempt is made to disentangle the laundry account for coals, gas, and labour from the general account, or the cost of soap, etc., used in this department from that used in cleaning. Thus the item of "washing" is mere guesswork, and no one has any real grip on the expenditure in this direction. More money slips unawares away in institutions than from deliberate mismanagement, and in no department is this truth more conspicuously exemplified than in the case of the laundry. In every hospital the cost of the washing per occupied bed ought to be an ascertained fact, and this cost ought to be compared one year with another, one hospital with another, until a good working average has been arrived at, which should not be overstepped without good reason shown.

At Guy's Hospital the cost of washing in the hospital is carefully calculated and is stated distinct from that of the nurses, which amounts to £4 3s. per head. It works out at an average of £3 a year per occupied bed. Taking the total at the Middlesex, and deducting £6 per head for nurses' laundry, the cost works out at £4 per bed. At the London Hospital, making the same deduction, the cost is £3. At the Royal Free, where, as in the other hospitals named, there is a private laundry, the cost is considerably less, not exceeding £2 a bed, without making any deduction for the nurses' laundry. In hospitals out of London it is the exception to find any comprehensive estimate attempted of the cost of washing. At the Leicester Infirmary, however, a valuable statement is supplied of the entire cost of the laundry. We quote it as an admirable example of the manner in which such accounts should be presented, and we do not doubt that the good policy of making a clear statement of cost in this direction will have the desired effect of inducing subscribers to come forward and undertake the cost of urgently needed alterations to the infirmary laundry.

| | £ | s. | d. |
|---|---|---|---|
| Washing and cleaning materials ... ... ... | 115 | 7 | 4 |
| Water, fuel and lighting ... ... ... ... | 67 | 2 | 7 |
| Wages of laundry superintendent, engineer, etc. | 270 | 18 | 2 |
| Renewals and repairs to machinery and buildings ... ... ... ... ... ... | 21 | 0 | 5 |
| Insurance, rates, taxes (proportion), cost of supervision, etc. ... ... ... ... ... | 40 | 0 | 0 |
| Total | 514 | 8 | 6 |

This works out at about £3 per occupied bed, making no deductions for the washing of the nursing staff, which is probably included. So far, then, as these scanty figures throw light on the subject, it would seem that £3 per occupied bed is a not unreasonable sum to reckon for the laundry in hospitals where great demand is made on the beds and where the atmosphere is tainted with smoke. We are not concerned with the question of how best and most economically to run a hospital laundry. That subject is complicated by many considerations touching the cost of fuel and the price of labour, with knotty points about the size of boilers and the best kind of machinery. Such matters often lie entirely outside the matron's jurisdiction. But she is directly concerned in the quantity of soiled linen sent out from the wards, and it is on this point that economy as well as efficiency needs to be taken into consideration. The economical aspect of the quantities despatched to the laundry is, it will be fully admitted, not the most important. But just as good catering is less expensive than bad, so the laundry bills of a matron who is intensely particular about the appearance of the beds and the cleanliness of everything used in the wards will probably be lower than those of the hospital where things are done under no particular system, and each sister is a law to herself. The matron who loves clean linen knows exactly what ought to be used in each department, and she takes some pains to see that her subordinates know how to keep matters up to the mark without extravagance. She makes allowance for the fact that a succession of medical students leaning over a bed will not improve the appearance of the upper sheet. She knows that when the wind is in a certain direction the smoke from a not too distant factory will invade the wards and call for additional linen. She knows when there has been unusual pressure on the beds of any particular ward, and will take steps to see that the ordinary supplies are supplemented if necessary. And because she understands precisely what class of case will make demands on the linen-cupboard, she is able to detect when the ward sister is dealing lavishly with her supplies without good cause. It is, however, very difficult to apply an effectual check to what is used in each ward, because emergencies are the rule. The matron of a non-clinical provincial hospital writes: "On an average we use 80 sheets, 6 blankets, 36 pillow cases, 10 towels a week for 24 beds. The sheets are changed once a week and the blankets when required." The matron of a London clinical hospital writes: "A good weekly average for a double ward of 30 beds is 70 sheets, 45 draw sheets, and 45 pillow cases." The top sheets are commonly changed twice a week in ordinary cases, and the blankets once in six weeks. Circumstances vary so much as between one ward and another that it is impossible to lay down any hard and fast line for the guidance of the sister. This is not, however, to say that she ought not to be guided by clearly defined rules in this most important matter.

Under able management the outgoings in the matter of soiled linen from each ward are under observation, and extravagance is at once checked.

